# Loss of *akt-1* throughout development leads to abnormal chemosensory behaviors in *C. elegans*

**DOI:** 10.17912/micropub.biology.002473

**Published:** 2026-10-30

**Authors:** Meredith I Williams, Hope Msengi, Rachel Arey

**Affiliations:** 1 Center for Precision Environmental Health, Baylor College of Medicine, Houston, TX, United States; 2 Department of Neuroscience, Baylor College of Medicine, Houston, TX, United States; 3 Department of Pathology and Immunology, Baylor College of Medicine, Houston, TX, United States; 4 Department of Molecular and Cellular Biology, Baylor College of Medicine, Houston, TX, United States

## Abstract

The *C. elegans* AKT serine/threonine kinase AKT *-* 1 is widely studied because it is a molecular player in multiple ubiquitous signaling pathways. *akt-1* mutants have been associated with gross, organismal-level phenotypes. In addition to organismal physiology, pathways associated with *akt-1* are known to regulate neuronal plasticity and behavior; therefore, *akt-1* mutants could be valuable for behavioral characterization. Here, we report that *akt-1* loss-of-function mutants demonstrate perturbed chemotaxis behaviors which could confound experiments that rely on proper chemosensation. We also determined that adult-only administration of *akt-1* RNAi is sufficient to overcome these behavioral deficits, suggesting these defects are likely developmental in origin.

**Figure 1. Loss of akt-1 throughout development via mutation or RNAi knockdown causes defective olfactory chemotaxis that can be avoided with adult-only manipulations:** A) 0x outcrossed *akt-1(ok525)* mutants have abnormally low chemotaxis indices to 10% butanone, due to large percentage of worms distributed around the chemotaxis plate. p-value= <0.0001. n = 15 assay plates. B) Schematic of *akt-1* phenotype observed during the chemotaxis assay. C,D). After 5 rounds (5x) of outcrossing, abnormal chemotaxis behaviors are observed towards strong attractants 0.1% butanone (C) and 1% diacetyl (D) in *akt-1(ok525)* mutants. p-value= <0.0001. n = 15 assay plates. E) Schematic of *akt-1(ok525)* allele and new *akt-1(ary2)* allele generated by CRISPR. F,G). *akt-1(ary2)* worms exhibit same abnormal chemotaxis behaviors as *akt-1(ok525)* animals towards strong attractants 0.1% butanone (F) and 1% diacetyl (G). p-value= <0.0001. n = 15 assay plates. H) Schematic of timelines of different RNAi administration to knockdown *akt-1* in specific tissues and different developmental timepoints. I,J). Whole-life administration of *akt-1* RNAi results in abnormal chemotaxis behaviors towards strong attractant 0.1% butanone in both wild-type (I, p-value = 0.0012) or neuronally-sensitive *sid-1 [qt9] V; sqls71[rgef-1::GFP + rgef-1p::sid-1]* (J, p-value<0.001) animals. n = 15 assay plates. K). Wild-type animals receiving adult-only *akt-1* RNAi administration no longer display severely abnormal chemotaxis behaviors towards 0.1% butanone. p-value of worms on plate = 0.0490. n = 15 assay plates. L,M) Whole-life administration of *akt-1* RNAi results in abnormal chemotaxis behaviors towards strong attractant 1% diacetyl in both wild-type (L, p-value < 0.0013, <0.0001) or neuronally-sensitive *sid-1 [qt9] V; sqls71[rgef-1::GFP + rgef-1p::sid-1]* (M, p-value<0.0001) animals. n = 15 assay plates. N) Wild-type animals receiving adult-only *akt-1* RNAi administration no longer display severely abnormal chemotaxis behaviors towards 1% butanone diacetyl. n = 15 assay plates.

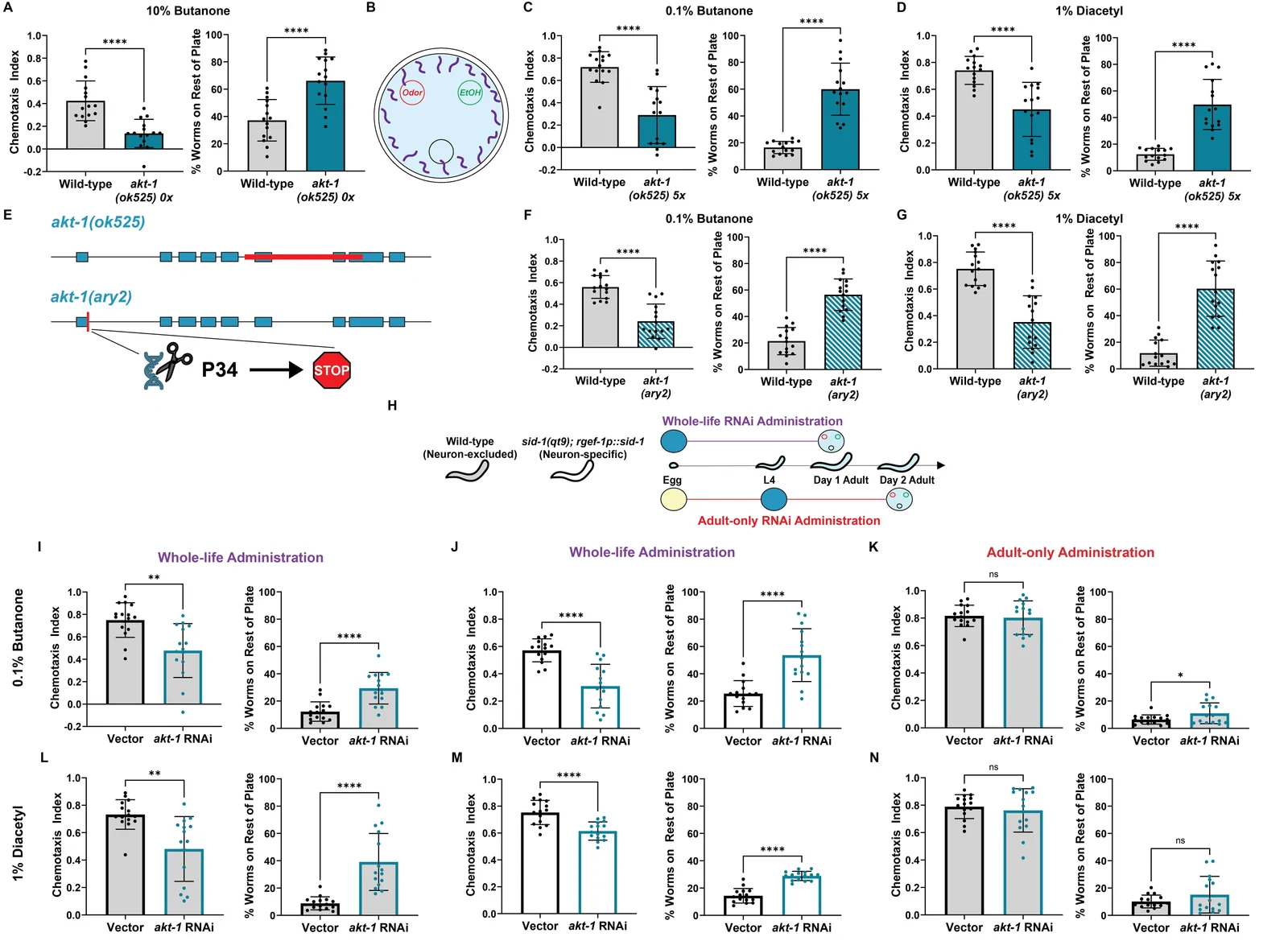



## Description

The *Caenorhabditis elegans* AKT-1 serine/threonine kinase ortholog, AKT-1 , is an extensively studied gene, with 39 distinct phenotypes reported from 87 independent studies (Blackwell et al., 2019; Sternberg et al., 2024; Alliance of Genome Resources Consortium 2024). Both loss-of-function (LoF) mutants and RNAi-based knockdown have been used to study the multifaceted effects of reduced *akt-1* function. These manipulations lead to many altered *,* gross, organismal-level phenotypes including changes in reproduction, movement, and lifespan (Sternberg et al., 2024). AKT-1 also plays a role in both insulin and mTOR signaling, which have well-documented effects on neuronal plasticity and behavior across species (Hoeffer and Klann 2010; Graber et al., 2013; Lee 2015; Borrie et al., 2017). The body of work examining the role of AKT-1 in behavior in the worm is presently much smaller than other organismal phenotypes. Previous studies have found that *akt-1* mutants exhibit deficits in salt chemotaxis and an increase in risk-taking behaviors (Tomioka et al., 2006; Jiu et al., 2010). However, an important consideration for behavioral studies is that mutants should not exhibit phenotypes that could confound interpretation of changes in behavioral outputs. For chemosensation-based assays, such as those that measure olfactory adaptation and learning and memory, perturbations in a worm's ability to detect an odor may be perceived as a learning or memory deficit rather than impaired olfaction (Colbert and Bargmann 1995; Stetak et al., 2009; Kauffman et al., 2010). This is an important distinction because unreported phenotypes, like chemosensory abnormalities, could lead to false positive results in future studies. Here we report that the commonly used *akt-1 ( ok525 )* mutant demonstrates previously unreported perturbed chemotaxis behaviors that persist even in a new CRISPR LoF line, which limits the usefulness of *akt-1* mutants for many behavioral studies.

**Mutation of *akt-1* leads to defective chemotaxis**

To examine the chemotactic ability of *akt-1 ( ok525 )* mutants, we used well-established, population-based assays where worms are exposed to an odorant that they have never encountered on an agar plate (Bargmann et al., 1993). While conducting preliminary chemotaxis (CTX) assays using a neutral concentration of butanone (10%), we observed that *akt-1 ( ok525 )* animals that had not been outcrossed (0x) displayed a deficit compared to control **(1A-left)** . We found that this deficit was due to animals not choosing either odor, represented by a high percentage of animals left on the plate **(1A-right)** , and instead crawling towards the sides and walls of the plate **(1B)** . To determine if the deficit was the result of background mutations or due to the presence of the *akt-1* mutation, we outcrossed *akt-1 ( ok525 )* animals with wild-type worms five times [5x *akt-1 ( ok525 )* ]. We then tested their chemotaxis towards two attractive odors - an attractive concentration of butanone (0.1%, **1C** ) and diacetyl (1%, **1D** ) to examine this phenotype more robustly. We chose these odorants because they require different sensory neurons, with attractive butanone chemosensation requiring the AWC neuron while attractive diacetyl chemosensation requires the AWA neuron (Bargmann et al., 1993; Wes and Bargmann 2001). We observed deficits in the naïve CTX of the 5x *akt-1 ( ok525 )* line to both odorants and concomitant increases in percent animals left on the plate, suggesting a broad effect involving many neurons and potentially multiple tissues. Since the deletion in the *akt-1 ( ok525 )* line is very large **(1E)** , we decided to create a more targeted LoF mutant using CRISPR/Cas9 (Evans 2006; Katic and Großhans 2013; Paix et al., 2015; Dickinson and Goldstein 2016; Schwartz et al., 2021). Briefly, we inserted a stop cassette at the end of the first exon in *akt-1 ( ary2 )* **(1E)** and confirmed this edit via whole-genome sequencing (Dickinson and Goldstein 2016; Calarco et al., 2025). We then tested the *akt-1 ( ary2 )* strain's naïve CTX towards the same strong attractants and again observed a deficit to both odorants and an increase in the number of animals left on the plate **(1F-G)** , similar to the *akt-1 ( ok525 )* strain. This suggests that genetic loss of *akt-1 ,* irrespective of the mutation introduced, is sufficient to drive this abnormal chemotactic behavior.

**RNAi knockdown of *akt-1* reveals a likely developmental origin of chemotaxis deficits that can be prevented with adult-only RNAi administration**

We next sought to establish if knockdown of *akt-1* via RNAi could circumvent the abnormal chemotaxis phenotype through more temporal and spatial control of gene manipulation (Zhuang and Hunter 2012). We administered *akt-1* RNAi to wild-type animals, which excludes knockdown in neurons, and a neuron-specific RNAi line ( *sid-1 [ qt9 ] V; sqls71 [ rgef-1 ::GFP + rgef-1 p:: sid-1 ]* , Yang et al., 2024). This RNAi treatment was performed either for the whole-life (WL, from hatch) or in adulthood (adult-only, AO). These conditions would allow us to determine if timing or neuron-specific loss was sufficient to overcome the phenotypes we had observed in mutant strains **(1H)** (Zhuang and Hunter 2012; Yang et al., 2024). We first fed wild-type worms *akt-1* RNAi for their entire life, which would most closely mimic the mutant condition (excluding neurons) to see if the abnormal chemotaxis behaviors would persist. We found that RNAi against *akt-1* administered WL in a wild-type background recapitulated our LoF mutants and observed CTX deficits to both butanone **(1I)** and diacetyl **(1L)** . Since RNAi administration in a wild-type background excludes neurons and produced CTX deficits, we hypothesized that knockdown in only neuronal tissues might not have an effect on CTX behaviors. Utilizing a genetic, neuron-specific strain, we administered WL RNAi against *akt-1* and found that knockdown in neurons alone was sufficient to recapitulate the chemotactic deficits and subsequent increase in the percent of worms left on the plate **(1J, 1M)** . This suggests that *akt-1* plays an important developmental role in multiple tissues in influencing sensory neuron function and that loss of *akt-1* in this critical window manifests in altered adult behaviors. AO administration in previous studies has proven to be a successful way to interrogate a gene's effect on behavior apart from its role in regulating development (Kaletsky et al., 2016; Arey et al., 2019; Hayden et al., 2024; Qin et al., 2025). Interestingly, we found that AO administration of *akt-1* in a wild-type background had no detectable effect on chemotaxis to both attractive odorants and resulted in low amounts of worms left on the plate **(1K, 1N)** . These results indicate that the abnormal chemotaxis behaviors described are likely a result of *akt-1 's* influence on development as systemic AO knockdown did not produce similar behaviors.

**Limitations and future directions**

We have shown that loss of *akt-1* throughout development *,* through either genetic manipulation or RNAi, is sufficient to produce abnormal olfactory chemotaxis behaviors. We also found that AO RNAi administration is sufficient to uncouple the developmental effects of *akt-1* from its influence on chemotactic assays. Therefore, future studies investigating *akt-1* in phenotypes that include an olfactory sensory component outside the context of development should be sure to use carefully timed knockdown approaches. For this study, we did not employ the auxin-inducible degron (AID) system which is another method offering temporal specificity in knockdowns (Zhang et al, 2015; Ashley et al., 2021). We expect that an *akt-1* AID system will behave similarly to the AO RNAi administration line as long as the knockdown is induced after the completion of neuronal development.

## Methods

*General C. elegans strains and maintenance*

All *C. elegans* strains were kept at 20°C on either standard nematode growth medium (NGM) plates or NGM plates containing 50ug/mL Carbenicillin and 1.0mM IPTG, referred to as NGICs. Plates were seeded with OP50 *Escherichia coli* or HT115 bacteria expressing *akt-1* RNAi (Clone purchased from Horizon; Catalog #RCE1182-202292360) or vector RNAi. Animals were developmentally synchronized using an alkaline-bleach solution on a population of gravid adults for egg collection; eggs were then washed with M9 buffer solution three times before plating on NGMs or NGICs. A list of the strains used and generated in this study are listed in the reagents section below.

*Strain development*

*akt-1 ( ary2 )* was generated via CRISPR/Cas9 genome editing and the gRNA and HDR template are listed below. Briefly, a stop codon cassette was inserted at the end of the first exon. The CRISPR/Cas9 injection, screening, and confirmation genotyping were conducted according to previous work. Whole genome sequencing was conducted through the University of Minnesota Model Organism Sequencing Core and confirmed our desired edit and no off-target effects.

gRNA (+ strand):

ACAUUCGAAACUGGAGACCAGUUUUAGAGCUAUGCU

HDR (- strand):

G*T*G GTA ATA TAT GAT CAG TAC CGA TAG AAA AAT CAA ACC TGC TAG CTT ATC ACT TAG TCA CCT CTG CTC TGG ACA AAC ТТС ССС ТАТ СТC CAG TTT CGA ATG TGC ТСТ ССТ ТTC TTG TGA AGC C*A*T

*RNAi Treatment*

For whole-life RNAi exposure, bleached eggs were immediately plated onto NGICs seeded with HT115 bacteria expressing either vector or *akt-1* RNAi (Horizon) and tested for behavior on day one of adulthood. For adult-only RNAi exposure, bleached eggs were plated onto NGM plates seeded with OP50 *E. coli.* At the L4 stage, worms were washed three times with M9 buffer solution before being transferred to seeded NGICs. Worms were exposed to RNAi for 48 hours before testing on day two of adulthood.

*Naïve Chemotaxis Assay*

Chemotaxis assays were performed as previously described (Bargmann et al., 1993). Briefly, assays were performed on unseeded 100mm NGM plates with two circles on opposite sides drawn approximately 5mm from the edge (See **1B** for illustration). 1uL of sodium azide was placed in the middle of both circles and dried before adding 1uL of odorant diluted in ethanol to the top left circle and 1uL of ethanol to the top right circle ( **1B** ). Well-fed, synchronized adult populations were washed into a conical tube using M9 buffer solution and washed three times to remove any residual food. Using a wide-bore pipette tip, worms were then placed near the bottom center circle ( **1B** ), or origin, of the plate and allowed to chemotax for an hour. Chemotaxis indices were calculated per plate as follows:

Chemotaxis Index= (# of worms on odorant - # of worms on ethanol) / (total # of worms)

We also reported the percent of worms that failed to choose either odorant as calculated per plate below:

Percent Worms on Rest of Plate= (# of worms _rest of plate_ )/[total # of worms _(odorant+ethanol+rest of plate)_ ] x 100

We define “rest of plate” as worms outside the odorant circles and origin (See **1B** for illustration). We tested the naïve preference to highly appetitive concentrations of butanone (0.1%) and diacetyl (1%) as previously reported. Each chemotaxis plate is represented as its own datapoint, with figures comprising five technical replicates per three independent populations for a total of 15 datapoints.

*Statistics*

All statistics were conducted with GraphPad Prism Software version 11.0.0. Welch's t-test was used to compare chemotactic indices and percent worms on rest of plate as it does not assume equal standard deviations. 

## Reagents

*Strain table*

**Table:** 

**Strain**	**Genotype**	**Source**
N2	Wild-type Bristol	CGC
MAH677	*sid-1 ( qt9 ) V; sqls71 [ rgef-1 p::GFP + rgef-1 p:: sid-1 ] *	CGC
RB759	*akt-1 ( ok525 ) *	CGC
5x RB759	*akt-1 ( ok525 )* Outcrossed with N2 five times	This study
RNA63	*akt-1 ( ary2 ) *	This Study
